# High Burden of Incidental and High-Grade Prostatic Carcinoma in Transurethral Resection of the Prostate (TURP) Specimens: A Retrospective Histopathological Analysis From Central India

**DOI:** 10.7759/cureus.100220

**Published:** 2025-12-27

**Authors:** Santosh Jayant, Amarnath Yadav, Abhiraj Ramchandani, Pankaj Gupta, Mahendra Singh Hora, Mamta Gupta, Maj Gen Shrikant Nema

**Affiliations:** 1 Pathology and Laboratory Medicine, Amaltas University, Dewas, IND

**Keywords:** benign prostatic hyperplasia (bph), gleason score, grade group, incidental adenocarcinoma, prostatic carcinoma/prostate cancer, prostatic intraepithelial neoplasia (pin), prostatic pathology, transurethral resection of the prostate (turp)

## Abstract

Introduction: The histopathological evaluation of transurethral resection of the prostate (TURP) chips, primarily performed for benign prostatic hyperplasia (BPH), remains critical for the incidental detection of pre-malignant and malignant prostatic lesions in resource-limited settings lacking comprehensive screening. This study aimed to characterize the histopathological spectrum of prostatic pathology and the severity of incidentally detected cancer in a rural area of Central India.

Methods: This was a single-center, retrospective study analyzing 218 consecutive TURP chip specimens received between July 2024 and November 2025. Data, including histopathological diagnosis, age distribution, and International Society of Urological Pathology (ISUP) 2014 Grade Group (GG) for incidental carcinoma, were collected. The Pearson Chi-square test was used to assess the association between age and malignancy.

Results: Out of 218 specimens, 75.68% (n = 165) were benign, and 18.36% (n = 40) were malignant. The prevalence of incidental prostatic adenocarcinoma (IPCa) was high at 17.90% (39 cases). Crucially, 53.8% (n = 21) of these adenocarcinomas were classified as aggressive (Grade Group 3 or higher), indicating a substantial burden of intermediate-to-high-grade disease. A statistically significant association was found between age and the presence of malignancy (Chi-square = 12.06, df = 4, p = 0.017). The highest proportional malignancy rate (62.5%) was noted in the youngest age group (<50 years).

Conclusion: The exceptionally high prevalence of incidental carcinoma and the significant proportion of high-grade disease (GG ≥ 3) underscore the necessity of mandatory and thorough histopathological evaluation of all TURP specimens, irrespective of pre-operative clinical findings, especially considering the high malignancy rate in younger men (<50 years), to ensure timely diagnosis and aggressive management in this population.

## Introduction

The prostate is a glandular organ in an adult's body that is typically shaped like a pear and weighs around 20 grams [1]. It is a retroperitoneal organ that surrounds the neck of the bladder and urethra. It is an exocrine gland and plays a major role in the production of seminal fluid. The prostatic tissue in adults can be categorized into four distinct zones: peripheral, central, transitional, and periurethral zones, each with unique biological and anatomical characteristics. Histologically, it is composed of glands that have basal cuboidal cells and inner secretory columnar cells arranged in a double-layered structure [1]. A majority of patients come in with concerns about urination and involuntary leakage. Among the conditions that impact the prostate gland, the most commonly seen in medical practice include benign prostatic hyperplasia, prostate cancer, and prostatitis [2].

Histopathological examination plays a crucial role in diagnosing and treating prostate lesions due to the similar clinical presentations of benign and malignant growths. Benign prostatic hyperplasia is the most frequently occurring non-cancerous prostate condition among men above 50 years old, and it exhibits significant differences in occurrence and fatality rates based on race and location [3]. The occurrence rate of this illness is just 8% in the fourth decade, yet it jumps to 50% in the fifth decade and rises to 75% in the eighth decade. Benign prostatic hyperplasia (BPH) is not a precancerous condition for prostate cancer, but it could be connected to prostate cancer that develops in the transition zone, according to Bostwick et al. (1992) [4]. In India, prostate cancer accounts for about 5% of all cancers in men [5,6]. Assessing prostatic abnormalities involves measuring the levels of serum prostate-specific antigen (PSA), digital rectal examination, and transrectal ultrasound, which are commonly used; however, biopsy is still considered the most reliable method for making a final diagnosis. Before the era of PSA testing, around 27% of prostate cancer cases were discovered by chance during transurethral resection of the prostate (TURP) procedures [7].

Consequently, samples from the prostate have posed a significant challenge for pathologists, with TURP specimens and prostate biopsies frequently presenting a diagnostic challenge to pathologists in practice. The current investigation was conducted to provide a comprehensive analysis of the histopathological spectrum of prostatic lesions encountered in TURP specimens. The specific aims of this study were to characterize the histopathological spectrum of prostatic lesions (benign, premalignant, and malignant) in TURP specimens, categorize incidental carcinomas using the International Society of Urological Pathology (ISUP) 2014 Grade Group system, and determine the statistical association between patient age and the presence of malignancy. This study also aimed to provide essential local data that contributes to a better understanding of prostate pathology.

## Materials and methods

This was a single-center, retrospective, observational study conducted in the Department of Pathology at Amaltas Institute of Medical Sciences, Dewas, Madhya Pradesh. The study analyzed 218 consecutive TURP chip specimens received for histopathological examination over 17 months, from July 2024 to November 2025. Inclusion criteria were all prostate tissue chips obtained via TURP from patients of any age presenting with lower urinary tract symptoms (LUTS), while prostatectomy specimens, core needle biopsies, and repeat TURP specimens from patients with a known pre-existing PCa diagnosis were excluded. The entire volume of the TURP chip specimens was meticulously grossed, processed, fixed in 10% neutral buffered formalin, embedded in paraffin wax, and sections were cut at 3-5 micron thickness. Sections were stained with hematoxylin and eosin (H&E) stain, and all malignant lesions were graded using the ISUP 2014 Grade Group (GG) system [8]. Data collected included the final histopathological diagnosis, patient age, and ISUP GG for incidental prostatic adenocarcinoma. Descriptive statistics (frequencies and percentages) and analytical statistics (Pearson chi-square test) were used to assess the prevalence and the association between age and malignancy, with a P-value < 0.05 considered statistically significant.

## Results

**Figure 1:**
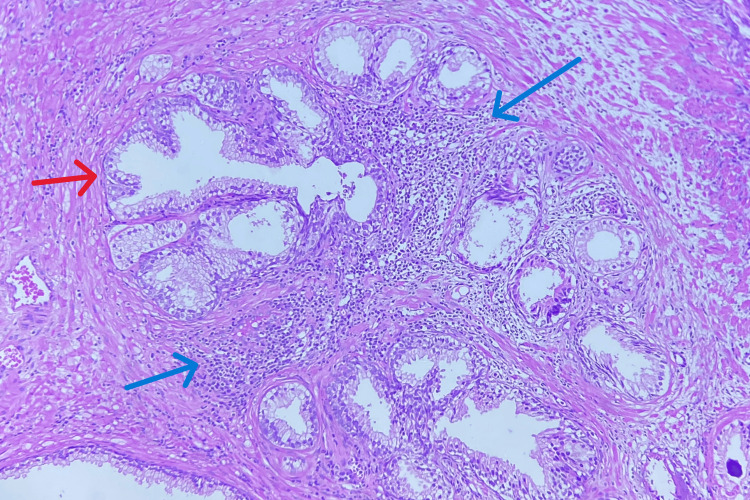
Benign prostatic hyperplasia with prostatitis The blue arrow shows associated chronic inflammation, and the red arrow shows the benign prostatic gland.

**Figure 2:**
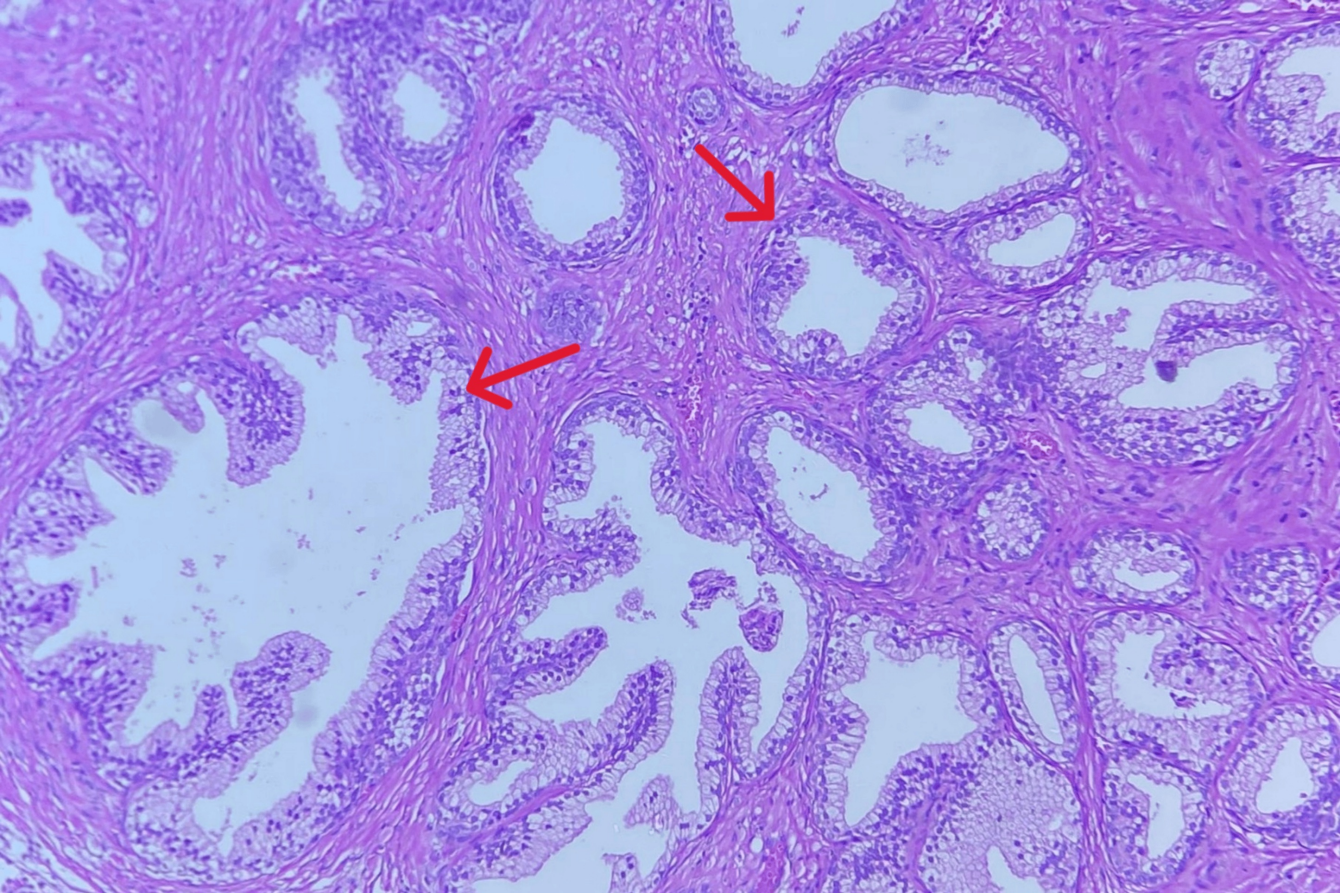
Benign prostatic hyperplasia The red arrow shows the benign prostatic gland.

The overall findings and histopathological spectrum of a total of 218 TURP specimens were analyzed. The majority of the specimens were categorized as benign, accounting for 75.68% (n = 165) of the total cases. Malignant and premalignant lesions accounted for 18.36% (n = 40) and 5.96% (n = 13) of the cases, respectively. Among the benign findings, BPH with associated chronic prostatitis was the single most common diagnosis, accounting for 38.53% (n = 84) of all cases (Figure 1). This was closely followed by BPH alone at 37.15% (n = 81; Figure 2).

**Figure 3:**
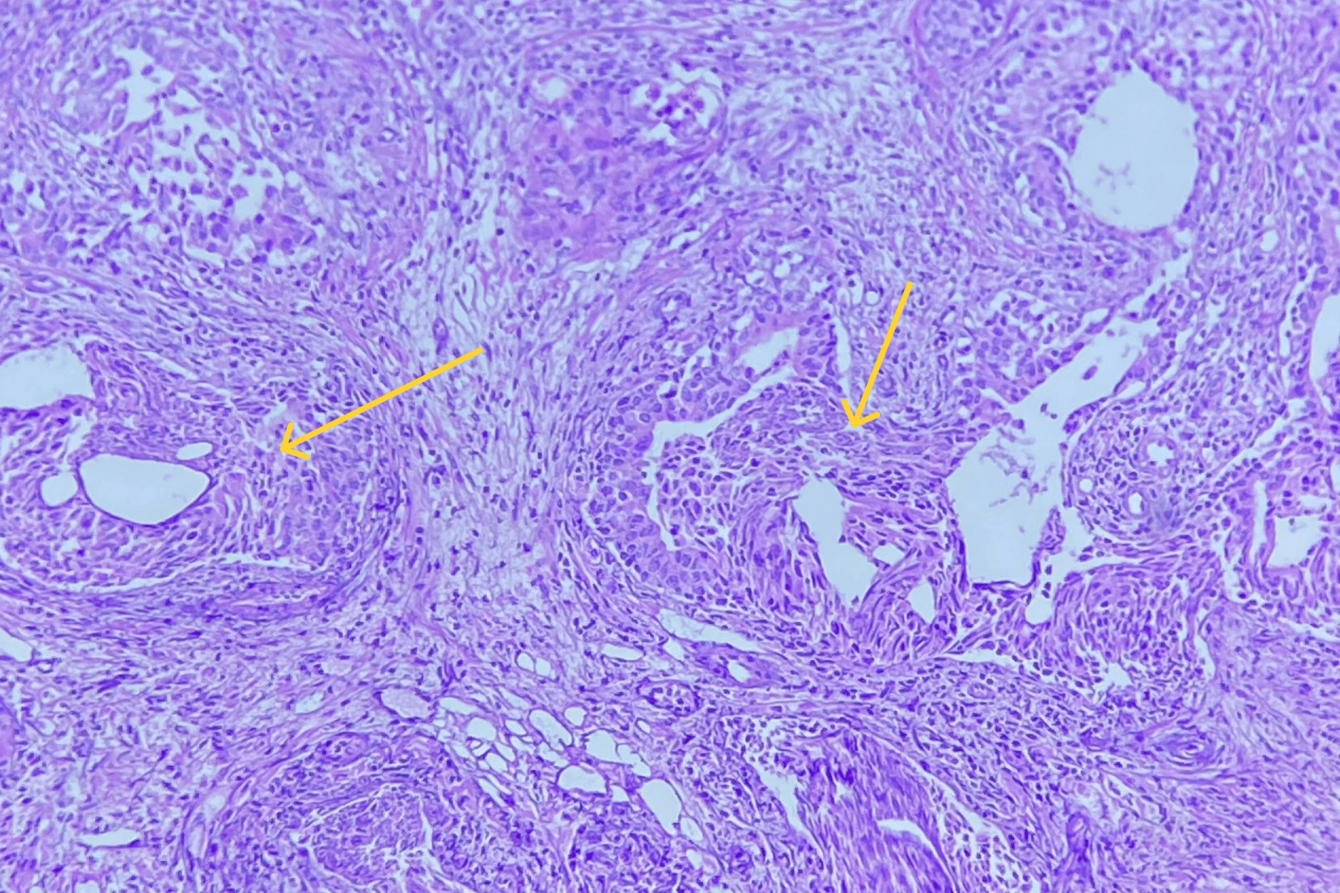
Prostatic intraepithelial neoplasia (PIN) The yellow arrow shows tufted pattern, hyperchromatic nuclei, and nucleolar prominence.

Premalignant lesions, comprising low-grade prostatic intraepithelial neoplasia (LGPIN) and high-grade prostatic intraepithelial neoplasia (HGPIN), were identified in 1.38% (n = 3) and 4.58% (n = 10) of the cases, respectively (Figure 3).

**Figure 4:**
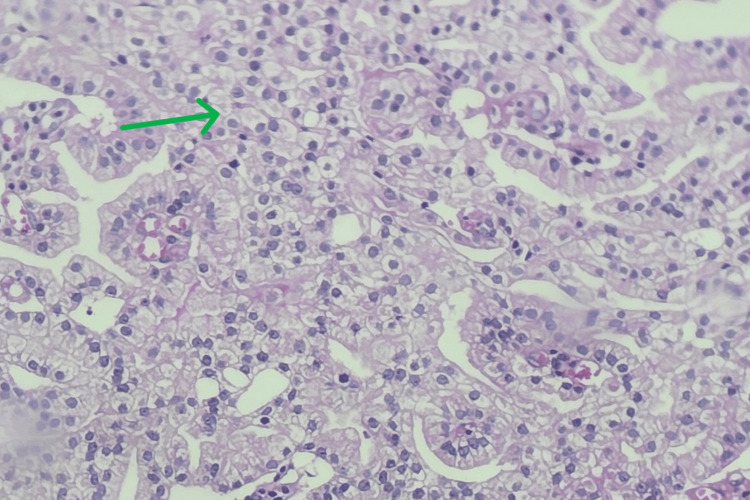
Prostatic adenocarcinoma (Gleason Grade Group 5) The green arrow shows sheets of cancer cells.

**Figure 5:**
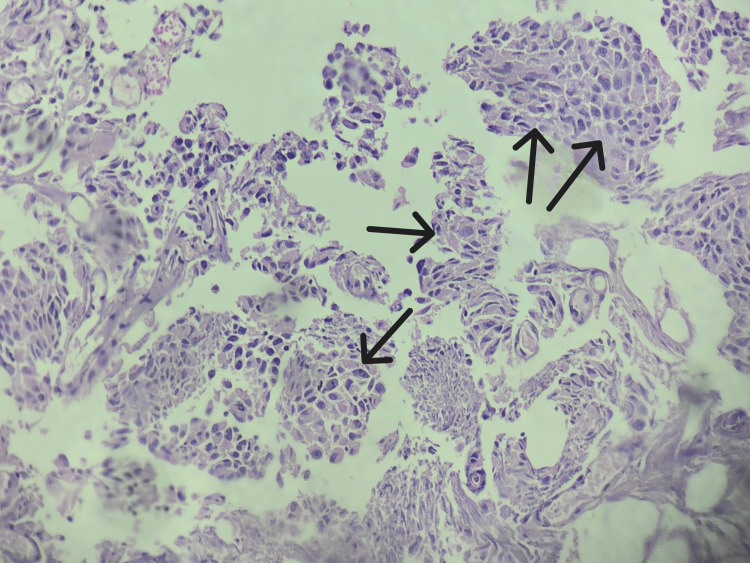
Transitional cell carcinoma of prostate The black arrow shows extensive proliferation of transitional cells with the presence of transitional malignant cells arranged in a multilayer pattern.

The prevalence of incidental adenocarcinoma was 39 cases (17.90%; Figure 4). There was one case showing features of transitional cell carcinoma lesion (0.46%; Figure 5).

The complete histomorphological spectrum is summarized in Table 1.

**Table 1:** Histomorphological spectrum in TURP specimens TURP: Transurethral resection of the prostate.

Pathological Classification	Specific Diagnosis	Number of Cases	Percentage of Total
Benign	Benign prostatic hyperplasia (BPH)	81	37.15%
	BPH with prostatitis	84	38.53%
	Subtotal benign	165	75.68%
Borderline/Premalignant	PIN low grade (LGPIN)	06	2.75%
	PIN high grade (HGPIN)	07	3.21%
	Subtotal borderline	13	5.96%
Malignant	Incidental prostatic adenocarcinoma	39	17.90%
	Transitional cell carcinoma	01	0.46%
	Subtotal malignant	40	18.36%
Total		218	100.00%

The age-wise distribution of cases (Table 2) showed that the majority of patients undergoing TURP were concentrated in the 70-79 years age group (34.86%, n = 76). The highest absolute number of malignant cases (n = 16) was also observed in this age group.

**Table 2:** Age-wise distribution of prostatic lesions

Age (in Years)	Benign Lesions	Malignant Lesions
<50	03	05
50-59	24	05
60-69	59	13
70-79	77	16
>80	15	01
Total	178	40

A Pearson chi-square test was performed to assess the relationship between age and malignancy. This analysis yielded a chi-square value of 12.06 (df = 4) and a P-value of 0.017, confirming a statistically significant association between the patient's age group and the presence of malignancy. Notably, the highest proportion of malignancy (62.5%, five out of eight cases) was recorded in the youngest age group (<50 years), compared to a 21.05% malignancy rate in the 70-79 age group, indicating a potentially more aggressive presentation in younger patients.

The 39 cases of incidental prostatic adenocarcinoma were graded using the ISUP 2014 GG system (Table 3). The analysis revealed a high proportion of aggressive tumors. A total of 53.8% (n = 21) of the cancers were classified as intermediate-to-high grade (GG ≥ 3).

**Table 3:** Distribution of cases of incidental prostatic carcinoma according to Gleason’s score and grade group

Grade Group	Gleason Score	Primary + Secondary Pattern	Total Cases
1	6	3 + 3	09
2	7	3 + 4	09
3	7	4 + 3	05
4	8	4 + 4	09
		3 + 5	00
		5 + 3	01
5	9/10	4 + 5	03
		5 + 4	01
		5 + 5	02

The most prevalent GGs were GG 1 (Gleason 3 + 3 = 6) and GG 2 (Gleason 3 + 4 = 7), each accounting for nine cases (23.1% each). However, GG 4 (Gleason 8) was the third most frequent category, comprising 10 cases (25.6%), demonstrating a significant number of high-risk tumors detected incidentally. The GG 5 (Gleason 9/10) cancers accounted for six cases (15.4%).

## Discussion

A study of 218 TRUP specimens was analyzed in the histopathology section, results were noted, and a comparison with other studies was undertaken as follows. The prevalence of IPCa in this study (Study A) is exceptionally high, recorded at 17.90% (39 cases out of 218). This figure notably exceeds the rates found in the comparative regional literature, which typically cluster around 10% to 14.5%, such as the 10.06% reported by Roşu et al. (2025) [9], 10.0% by Nayak et al. (2024) [10], and 14.4% by Rajani et al. (2020) [11]. The prevalence in the present study also exceeds the 10.3% reported in the Tanzanian study by Gunda et al. (2018) [12] and the 14.19% reported by Sujatha et al. (2019) [13] in a similar Indian study. This significantly elevated IPCa prevalence suggests that in the specific population under investigation, a substantial number of clinically silent cancers are diagnosed only after surgical intervention, highlighting the critical role of mandatory histopathology for all TURP specimens in resource-limited settings.

A crucial point of distinction for the findings of the present study is the high proportion of aggressive disease. Study A reported that 53.8% (21/39) of incidental adenocarcinomas were classified as aggressive (ISUP GG 3 or higher), which is a higher proportion of high-grade disease compared to what is commonly cited in the literature. For example, Roşu et al. (2025) [9] and Poste et al. (2025) [14] both found that the most frequent incidental cancer was the intermediate grade, Gleason score 7 (GG 2). Furthermore, a study focusing on younger men (≤65 years) by Perera et al. (2016) [15] found that only 30% of their cancers had a Gleason score ≥ 7. The predominance of high-grade disease in the present study implies that the incidentally discovered cancers in this cohort are, on average, more biologically aggressive and potentially carry a poorer prognosis, requiring careful management post-diagnosis.

While the majority of absolute cancer cases in all studies, including this study, are concentrated in the older age groups (typically 70-79 years), Study A highlights a unique proportional risk. We found that the highest proportional malignancy rate (62.5%) was observed in the youngest age group (<50 years). This contrasts with the general observation that cancer detection rates are higher in older patients undergoing TURP (e.g., a rate of 28.7% in the older group vs 13.4% in the ≤65 group, Parera et al. (2016) [15]). The striking finding of the highest relative risk in men under 50 suggests that for those who present with BPH symptoms severe enough to warrant TURP at a young age in this clinical setting, an underlying carcinoma should be considered with greater urgency than in most comparative populations.

The collective data confirm that BPH is the universal primary diagnosis in TURP specimens, as expected. The associated finding of prostatitis (chronic inflammation) is also a common feature across the literature, supporting the understanding that inflammation is a critical co-existing element in the pathophysiology of prostatic diseases. The finding of BPH with associated prostatitis in 38.53% of cases is consistent with the wide range reported by others, such as 50.4% reported by Nayak et al. (2024) [10], 55.70% by Roşu et al. (2025) [9] , and 46.3% by Poste et al. (2025) [14]. This consistency underscores the widespread recognition of chronic inflammation as a potent contributing factor in the development and progression of BPH.

Limitation

Missing Clinical Data

The study did not correlate histopathology with crucial clinical parameters such as preoperative PSA levels, prostate volume, or clinical staging (e.g., T1a vs. T1b), which would have provided richer prognostic information. Future studies are warranted to correlate these histopathological findings with patient-specific clinical data to better guide prognosis and individualized management decisions.

## Conclusions

Critically, 53.8% of these cancers were high grade (Grade Group 3 or higher), indicating a substantial burden of intermediate-to-high-grade disease at detection. The statistically significant association between age and malignancy, coupled with the concerningly high proportional malignancy rate observed in patients under 50 years, strongly emphasizes the need for mandatory and thorough histopathological evaluation of all TURP specimens. These results contribute essential regional epidemiological data on prostate pathology in Central India, guiding clinicians toward earlier diagnosis and more aggressive management of incidentally detected high-grade disease. We recommend that the clinical stage of LUTS and PSA levels be provided to the pathologist for better and more accurate diagnosis and grading of tumors.
